# Catchment Legacies and Time Lags: A Parsimonious Watershed Model to Predict the Effects of Legacy Storage on Nitrogen Export

**DOI:** 10.1371/journal.pone.0125971

**Published:** 2015-05-18

**Authors:** Kimberly J. Van Meter, Nandita B. Basu

**Affiliations:** 1 Department of Earth & Environmental Sciences, University of Waterloo, Waterloo, ON, Canada; 2 Department of Civil and Environmental Engineering, University of Waterloo, Waterloo, ON, Canada; CAS, CHINA

## Abstract

Nutrient legacies in anthropogenic landscapes, accumulated over decades of fertilizer application, lead to time lags between implementation of conservation measures and improvements in water quality. Quantification of such time lags has remained difficult, however, due to an incomplete understanding of controls on nutrient depletion trajectories after changes in land-use or management practices. In this study, we have developed a parsimonious watershed model for quantifying catchment-scale time lags based on both soil nutrient accumulations (biogeochemical legacy) and groundwater travel time distributions (hydrologic legacy). The model accurately predicted the time lags observed in an Iowa watershed that had undergone a 41% conversion of area from row crop to native prairie. We explored the time scales of change for stream nutrient concentrations as a function of both natural and anthropogenic controls, from topography to spatial patterns of land-use change. Our results demonstrate that the existence of biogeochemical nutrient legacies increases time lags beyond those due to hydrologic legacy alone. In addition, we show that the maximum concentration reduction benefits vary according to the spatial pattern of intervention, with preferential conversion of land parcels having the shortest catchment-scale travel times providing proportionally greater concentration reductions as well as faster response times. In contrast, a random pattern of conversion results in a 1:1 relationship between percent land conversion and percent concentration reduction, irrespective of denitrification rates within the landscape. Our modeling framework allows for the quantification of tradeoffs between costs associated with implementation of conservation measures and the time needed to see the desired concentration reductions, making it of great value to decision makers regarding optimal implementation of watershed conservation measures.

## Introduction

High levels of nonpoint source pollution associated with current agricultural practices have contributed to water quality impairment and destruction of aquatic ecosystem habitats at both local and global scales [1,2]. In particular, increased nutrient loads delivered from watersheds due to agricultural intensification, industrialization, and urbanization have led to the persistence of large hypoxic zones in both inland and coastal waters [3–7]. Watershed management practices to target these non-point source pollutants have in many cases resulted in little or no improvement in water quality, even after extensive implementation of conservation measures [8–10]. The *lag time* between implementation of conservation measures and resultant water quality benefits has recently been recognized as an important factor in their “apparent” failure [8,11]. Conservation measures are often implemented, however, without explicit consideration of such lag times, and with the expectation that they will lead to immediate benefits. Failure to meet such expectations then discourages vital restoration efforts [8]. In order to address this problem, it is important to quantify the lag times associated with watershed management efforts a priori and to implement restoration strategies that are targeted specifically at minimizing lag times as well as maximizing restoration benefits.

The focus of the present research is to develop a framework for understanding the time lags between land-use change or implementation of conservation measures and stream water quality benefits. We hypothesize that such time lags arise from legacies that have accumulated in the landscape over decades of human impact [12–14]. Legacies can be conceptualized as hydrologic legacy, in the form of dissolved solute that is delayed in its transport to the stream due to slow groundwater transport pathways, and biogeochemical legacy, arising from solute that has undergone biogeochemical transformation and that is retained within the soil matrix. Both solute and watershed attributes define whether such legacy sources will be created, and, if created, their spatial location within the watershed.

In the present study, we focus specifically on the fate of anthropogenic nitrogen (N) in predominantly agricultural watersheds. Significant time lags between land-use change and the expected decreases in stream nitrate concentrations have consistently been noted [8]. Such time lags have chiefly been attributed to what we have defined as the *hydrologic* N legacy, a legacy existing primarily in groundwater reservoirs or thick unsaturated zones in the form of dissolved nitrate [9,15,16]. Recent work, however, suggests that consideration of this hydrologic legacy alone does not adequately account for the magnitude of legacy N existing within intensively managed landscapes. Watershed-scale mass balance studies, for example, consistently indicate the presence of "missing" N stores [17–24], and recent modeling of the global N cycle has led to estimates of terrestrial N sequestration ranging from 27–100 Tg N/yr [25–27]. At the plot scale, a recent isotopic tracer study designed to investigate the long-term fate of nitrate fertilizer has shown that approximately 15% of fertilizer N applied to agricultural land is present within the soil profile in organic form 30 years after its initial application [28]. In another study [29], isotopic data were used to demonstrate that the nitrate measured in streams is generated from organic nitrogen created from fertilizer applied to the landscape decades previously. These results are indicative of high levels of N retention within agricultural soil over a multi-year period, and thus the existence of a biogeochemical N legacy, which is corroborated by our recent research showing a basin-wide accumulation of organic N in the Mississippi River Basin [30].

Despite such studies demonstrating the existence of both hydrologic and biogeochemical N legacies, most mechanistic watershed models lack an explicit mechanism to describe the effects of these legacies on stream nitrate concentrations [8,31,32]. Most lumped watershed models such as SPARROW and GlobalNEWS as well as the Net Anthropogenic Nitrogen Inputs (NANI) mass balance approach assume the N cycle to be at a steady state, either on a yearly basis or over a multi-year period, such that stream export is a fixed percentage of net annual inputs [33–37]. Even mechanistic attempt at quantify the benefits of different pollution-reduction scenarios (e.g. land-use change, reductions in fertilizer application, etc.) using mechanistic models such as SWAT have focused only on the concentration reduction benefit that will be achieved at infinite time, with no consideration of the time that will be required to achieve that goal [38,39]. Such consideration, however, is critical for watershed managers who must make decisions regarding the allocation of limited resources for conservation [8].

In this paper, we develop a parsimonious analytical model to quantify the concentration reduction benefits associated with watershed restoration efforts as a function of both hydrologic and biogeochemical legacies, with particular attention being paid to the ways in which spatial patterns of landscape conversion impact concentration reduction scenarios. Concentration reductions are considered to occur as a function of both the groundwater travel time distribution and biogeochemical controls, including the existence of a biogeochemical N legacy within the soil profile and denitrification dynamics along groundwater pathways. The paper presents analytical relationships between: (a) percent reductions in mean concentrations at the watershed outlet as a function of the fractional watershed area over which the management practice is implemented, and (b) the temporal trajectory of watershed response that defines the time required to achieve required reductions in contaminant concentrations. Using these analytical relationships, we explore idealized scenarios of land-use conversion and compare these results with concentration trajectories observed in a small Midwestern watershed undergoing an extensive prairie habitat restoration project. We further use these relationships to establish an optimization framework for meeting concentration reduction goals by exploring tradeoffs between costs associated with the conversion of land out of row-crop production and the time required to achieve the desired concentrations. Such explorations enable analysis of the performance of conservation measures under spatially varying patterns of intervention as a function of legacy N accumulation, N removal dynamics in the subsurface, and watershed travel time distributions.

## Model Development

Our conceptual framework is based on the assumption that legacy nutrient stores are present within anthropogenic landscapes and lead to time lags between land-use change and improvements in water quality. Such nutrient legacies have developed in agricultural watersheds as a function of long-term application of N and phosphorus (P) fertilizers, with a strong linear correlation having been found between N and P levels in soils and multi-decadal cumulative nutrient surpluses [13,30,40]. Our focus herein is specifically on the N legacy in agricultural watersheds, but this approach could be readily adapted to other solutes.

**Fig 1:**
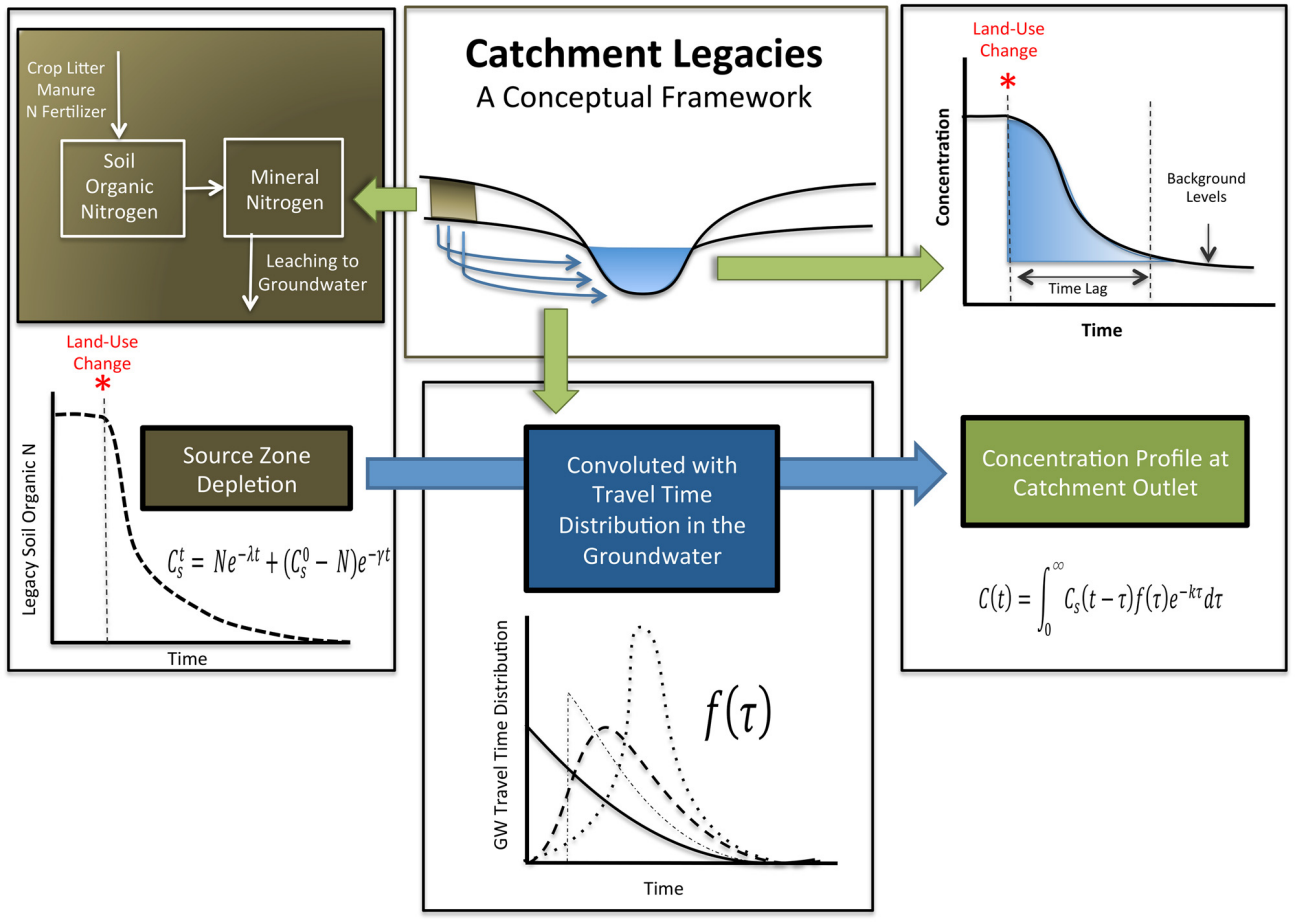
Conceptual framework for predicting catchment scale time lags as a function of hydrologic and biogeochemical legacies in the landscape. The left frame represents depletion of biogeochemical legacy in the source zone. The source zone depletion function is then convoluted with the groundwater travel time distribution (middle frame) to ultimately describe concentrations at the catchment outlet (right frame).

As shown in Fig 1, nutrient legacies produce an internal landscape memory, thus contributing to elevated stream nutrient concentrations for years after external nutrient loading is reduced or stopped altogether. In order to develop an expression for the concentration trajectory at the catchment outlet following land-use change, we conceptualize the landscape to be composed of a bundle of stream tubes, with each point on the landscape being associated with an individual stream tube characterized by a specific groundwater travel time to the catchment outlet [41]. The full amalgamation of points for the catchment leads to a specific groundwater travel time distribution for the catchment outlet, *f*(*τ*) This distribution, in turn, controls the concentration trajectory at the outlet, *C*(*t*) [42–44], as described by the following equation:

$$C(t)=\int_{0}^{\infty }C_{s}(t-\tau )f(\tau )e^{-k\tau }d\tau$$ (1)

where, *C*_*S*_(*t—τ*) is the contaminant input function or “source function” from the unsaturated zone, and *k* [T^-1^] is the first-order rate constant that describes removal processes in the aquifer.

The source function (Fig 1), developed in Section 2.1, is controlled by the biogeochemical legacy in the unsaturated zone, which for N is a function of both historic anthropogenic N inputs to the landscape and the rate of N depletion from such stores. Each point in the watershed is characterized by its particular source function, which changes form as a function of the timing of human interventions such as land-use change or implementation of conservation measures. While biogeochemical legacy is conceptualized using the “source function,” the hydrologic legacy is captured in the travel time distribution, which describes how the source concentrations are being modified as they travel through the watershed (Section 2.2). The resulting outlet concentration is a function of both the hydrologic and biogeochemical legacy, and the patterns of land-use change, as described in the following sections.

### 2.1 Biogeochemical Legacy and the Source Function

The left frame of Fig 1 provides a simple schematic for our model of biogeochemical legacy depletion within the source zone after conversion from row-crop agriculture to grassland. Within this framework, excess soil organic N, which has accumulated in response to long-term application of fertilizer N and which constitutes the biogeochemical N legacy, is mineralized to inorganic N, entering the soil mineral N pool. This inorganic N then leaches to groundwater, primarily in the form of nitrate. Although plant uptake and litter inputs will continue to occur after conversion to grassland, we consider these processes to be part of a baseline scenario and therefore only take into account dissipation of excess N through the leaching pathway. In our current simulations, we consider only scenarios in which landscape conversion results in a complete cessation of fertilizer application to the soil system, although this formulation can be easily modified to include cases with ongoing but reduced levels of fertilizer application.

Decomposition of soil organic matter is typically modeled as having first-order reaction kinetics, proportional to the amount of substrate to be decomposed [45,46]. Accordingly, within our modeling framework the excess (legacy) soil organic N (SON) is considered to decay over time as a first-order process with a rate constant *λ* (T^-1^), such that the mass of legacy N **(*M***_*SON*_ **)** at any time *t* is given by:

$$\frac{dM_{son}}{dt}=-\lambda M_{son}$$ (2)

The N leaving the SON pool enters the mineral N pool that leaches into the groundwater, such that at any point in time the concentration of dissolved N (C_s_; M/L^3^) can be described by the following equation:

$$\frac{d(V_{w}C_{s})}{dt}=\lambda M_{son}-QC_{s}$$ (3)

where, *Q* is the mean annual recharge and V_w_ (= *nsV*) is the water volume in the source zone, with *n* being the porosity, *s* the saturation and *V* the volume of the soil column per unit area within the source zone. Here, the first term on the RHS is the input from the organic pool and the second term is the loss from the source zone via leaching. Solving Eqs 2 and 3 leads to:

$$M_{son}=M_{son}^{0}e^{-\lambda t}$$ (4)

$$C_{s}^{t}=Ne^{-\lambda t}+(C_{s}^{0}-N)e^{-\gamma t}$$ (5)

where $N=\frac{\lambda M_{son}^{0}}{\left(Q-\lambda snV\right)}$;$\gamma =\frac{Q}{snV}$; $M_{son}^{0}$ is the initial mass of SON; $C_{s}^{0}$ is the initial concentration of nitrate within the source zone; and $C_{s}^{t}$ is the nitrate concentration at time *t* within the source zone, and acts as the source function described in Eq 1. The source function can thus be described as a Heaviside function, with $C_{s}^{0}$ being the initial steady-state concentration prior to initiation of land-use change, and $C_{s}^{t}$ describing the concentration trajectory after land-use change has been initiated.

### 2.2 Hydrologic Legacy and Patterns of Land-use Change

We define the hydrologic nutrient legacy as nutrients present in a dissolved form in both the saturated and unsaturated zones. Time lags associated with hydrologic legacy can range from days to weeks to hundreds of years as a function of the distance groundwater must travel to the catchment outlet, the physical properties of the underlying aquifer, and the gradient driving flow through the subsurface [9,47].

**Fig 2:**
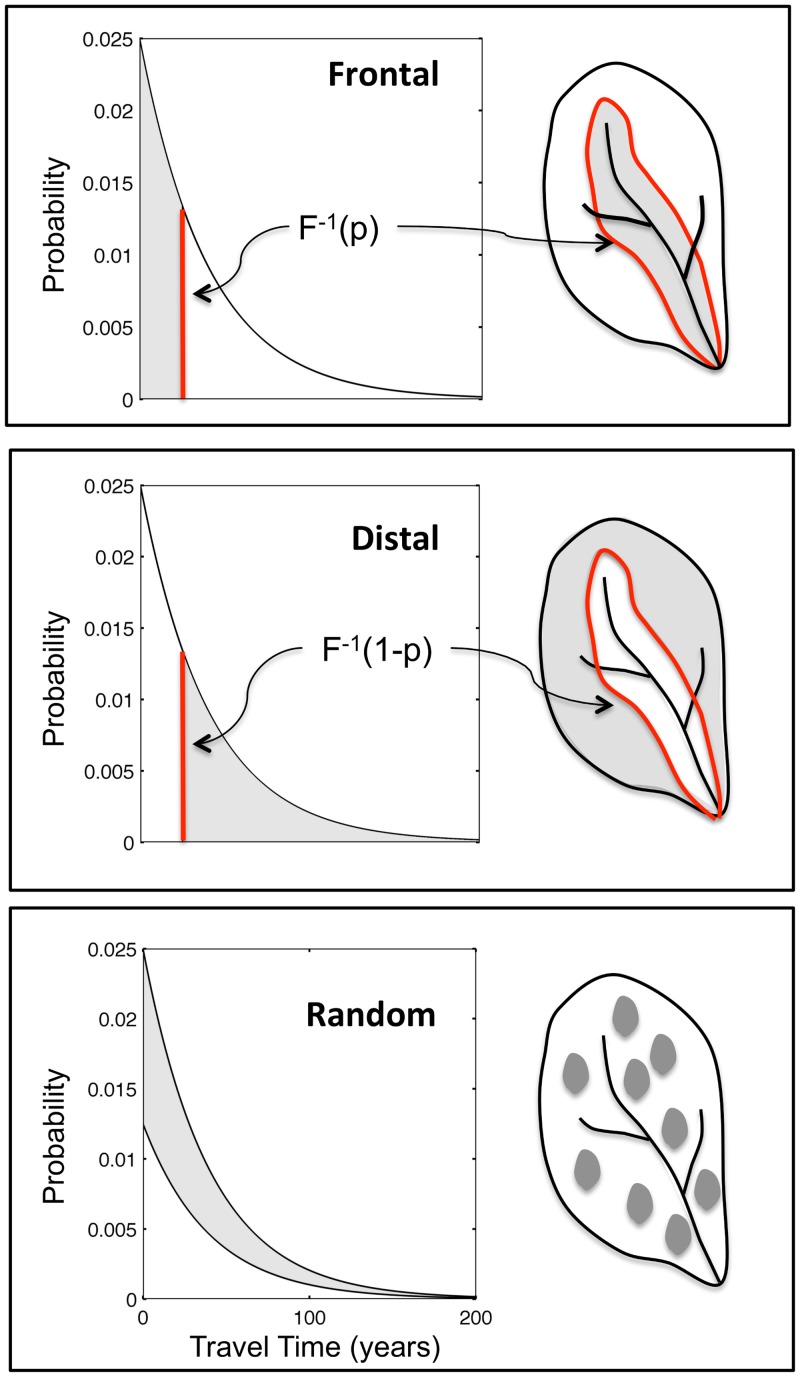
Conceptual framework showing spatial patterns of land-use change as truncations of the groundwater travel time distribution. The grey shaded areas correspond to the fractional areas (p) of the watershed over which land-use change has occurred for the (a) frontal, (b) distal and (c) random conversion scenarios. The red line in the frontal and distal scenarios is equal to the abscissa of the cumulative frequency distribution of travel times, corresponding to an ordinate of p (or 1-p for the distal scenario), and is the demarcation line between areas that have and that have not undergone land-use change.

Land-use change in a watershed leads to switching of the source function between $C_{s}^{0}$ and $C_{s}^{t}$. Theoretically, individual points in the landscape may be switched at different points in time, or not switched at all, leading to an infinite number of scenarios that are convoluted as in Eq 1, creating unique concentration trajectories at the outlet. Here, we conceptualize three end-member scenarios based on the distribution of travel times for the watershed. As shown in Fig 2, spatial patterns of land-use change can be described as truncations of the groundwater travel time distribution, with the three scenarios of change being: (a) frontal, (b) distal and (c) random. The frontal approach corresponds to scenarios where land parcels with the shortest travels times to the catchment outlet are preferentially converted and involves a left-to-right truncation of the exponential travel time distribution (Fig 2a), as indicated by the grey shaded area of the figure. Conversely, a distal approach corresponds to a preferential conversion of parcels with the longest travel times to the outlet and involves a right-to-left truncation (Fig 2b). The third approach, a random conversion, corresponds to a scenario where land-use change has occurred randomly throughout the catchment, with no correlation between land-use change and the groundwater travel time distribution (Fig 2c).

Equations for the flow-averaged concentrations at the outlet at any time *t* after initiation of land-use change, *C*_*ac*_(*t*;*p*) normalized by the concentration before the change *C*_*bc*_(*t*;*p*) for the three different spatial conversion scenarios, and a fractional land-use change p can be developed as follows: Frontal

$$\frac{c_{ac}}{c_{bc}}(t;p)=[\begin{array}{l} \frac{\int_{0}^{t}C_{s}^{t-\tau }f(\tau )e^{-k\tau }d\tau +\int_{t}^{\infty }C_{s}^{0}f(\tau )e^{-k\tau }d\tau }{\int_{0}^{\infty }C_{s}^{0}f(\tau )e^{-k\tau }d\tau },\;0\leq t<F^{-1}(p)\\ \frac{\int_{0}^{F^{-1}(p)}C_{s}^{t-\tau }f(\tau )e^{-k\tau }d\tau +\int_{F^{-1}(p)}^{\infty }C_{s}^{0}f(\tau )e^{-k\tau }d\tau }{\int_{0}^{\infty }C_{s}^{0}f(\tau )e^{-k\tau }d\tau },\;t\geq F^{-1}(p) \end{array}$$ (6)

Distal

$$\frac{c_{ac}}{c_{bc}}(t;p)=[\begin{array}{l} 1,\;0\leq t<F^{-1}(1-p)\\ \frac{\int_{0}^{F^{-1}(1-p)}C_{s}^{0}f(\tau )e^{-k\tau }d\tau +\int_{F^{-1}(1-p)}^{t}C_{s}^{t-\tau }f(\tau )e^{-k\tau }d\tau +\int_{t}^{\infty }C_{s}^{0}f(\tau )e^{-k\tau }d\tau }{\int_{0}^{\infty }C_{s}^{0}f(\tau )e^{-k\tau }d\tau },\;t\geq F^{-1}(1-p) \end{array}$$ (7)

Random

$$\frac{c_{ac}}{c_{bc}}(t;p)=\frac{\int_{0}^{t}pC_{s}^{t-\tau }f(\tau )e^{-k\tau }d\tau +\int_{0}^{t}(1-p)C_{s}^{0}f(\tau )e^{-k\tau }d\tau +\int_{t}^{\infty }C_{s}^{0}f(\tau )e^{-k\tau }d\tau }{\int_{0}^{\infty }C_{s}^{0}f(\tau )e^{-k\tau }d\tau }$$ (8)

Here *F* ^-1^()is the inverse cumulative distribution function of the groundwater travel time distribution. It represents the travel time associated with a specific fractional area of landscape conversion and acts as a dividing line (red line in Fig 2a and 2b) between areas of the watershed bringing in “converted” groundwater and areas bringing in “unconverted” groundwater. The above equations have been developed with the assumption that the groundwater travel time distribution is a complete distribution from 0 to infinity. In actuality, however, these distributions would be truncated, with the maximum travel time being defined by the size as well as the geomorphic characteristics of the watershed, and the equations can be easily modified for truncated distributions following Jawitz et al. [48].

Groundwater travel time distributions have been assumed to have multiple functional forms based on a range of model types, from the simplest piston-flow model, which assumes that all flow paths have the same velocity and path length, to a dispersion model based on a 1-D solution of the advection dispersion equation [44]. One of the simplest and most widely used forms is the exponential:

$$f(\tau )=\frac{1}{\mu }e^{-\frac{\tau }{\mu }}$$ (9)

where *τ* is the travel time and *μ* is the mean travel time for the watershed. Here, we have used the exponential distribution to develop algebraic expressions for the flow-averaged concentration after land-use change following the three different patterns of intervention described in Eqs 6,7, and 8.. Frontal

$$\frac{c_{ac}}{c_{bc}}(t;p)=\left\{ \begin{array}{l} c_{1}\frac{N}{C_{0}}(e^{-\lambda t}-e^{-\sigma t})+c_{2}\frac{\left(C_{s}^{0}-N\right)}{C_{s}^{0}}(e^{-\gamma t}-e^{-\sigma t})+e^{-\sigma t}\;0\leq t<F^{-1}(p)\\ c_{1}\frac{N}{C_{0}}(e^{-\lambda t}-e^{-F_{f}(k-\lambda +\frac{1}{\mu })-\lambda t})+c_{2}\frac{\left(C_{s}^{0}-N\right)}{C_{s}^{0}}(e^{-\gamma t}-e^{-F_{f}(k-\gamma +\frac{1}{\mu })-\gamma t})+e^{-F_{f}\sigma }\;t\geq F^{-1}(p) \end{array}\right.$$ (10)

*D*istal

$$\frac{c_{ac}}{c_{bc}}(t;p)=\left\{ \begin{array}{l} 1\;0\leq t<F^{-1}(1-p)\\ 1+c_{1}\frac{N}{C_{0}}(e^{-F_{d}(\sigma -\lambda )-\lambda t}-e^{-t(\sigma -\lambda )})+c_{2}\frac{\left(C_{s}^{0}-N\right)}{C_{s}^{0}}(e^{-F_{d}(\sigma -\gamma )-\lambda t}-e^{-t(\sigma -\gamma )})-e^{-F_{d}\sigma }+e^{-\sigma t}\;t\geq F^{-1}(1-p) \end{array}\right.$$ (11)

Random

$$\frac{c_{ac}}{c_{bc}}(t;p)=1-p(1-c_{1}\frac{N}{C_{0}}(e^{-\lambda t}-e^{-\sigma t})-c_{2}\frac{\left(C_{s}^{0}-N\right)}{C_{s}^{0}}(e^{-\lambda t}-e^{-\sigma t})-e^{-\sigma t})\;t\geq 0$$ (12)

where

$$\sigma =1+\mu k;\;c_{1}=\frac{1+\mu k}{1+\mu k-\lambda \mu };\;c_{2}=\frac{1+\mu k}{1+\mu k-\gamma \mu };$$

$$F_{f}=F^{-1}(p);\;F_{d}=F^{-1}(1-p)$$

Here, *F*_*f*_ and *F*_*d*_ represent the longest and shortest groundwater travel times, respectively, for land parcels that have undergone land-use conversion. Note that in the above formulations we have implicitly assumed that the watershed is homogeneous in terms of land use. This assumption is not, however, a limitation of the approach. For heterogeneous land use, the groundwater travel time distribution of interest is that of the areas contributing solute to the watershed outlet. For example, when only a fraction of the watershed area is under row crops and the management practice involves conversion of row crops to prairies, the travel time distribution used in Eq 9 would be that of the cells originally under row crop.

## Materials and Methods

### 3.1 The Walnut Creek Case Study

#### 3.1A Site Description

**Fig 3:**
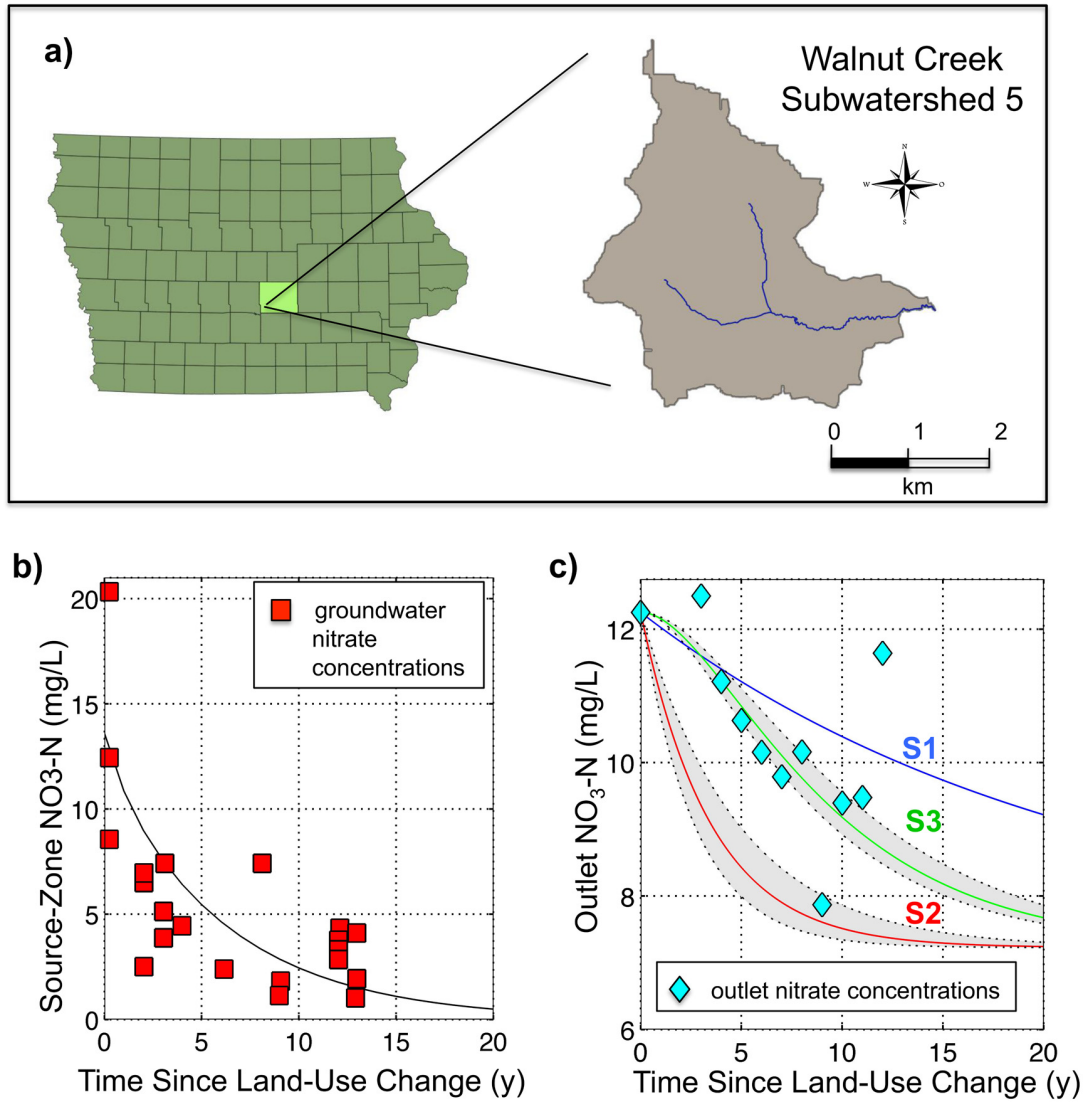
Site Information and Results for the Walnut Creek Case Study. (a) Subwatershed 5 (7.9 km^2^) of the Walnut Creek watershed, Jasper County, Iowa; (b) Data points correspond to groundwater nitrate concentrations in 19 monitoring wells across a chronosequence of restorations sites. Biogeochemical Legacy Depletion: Source Zone Nitrate-N Concentration as a function of time since land-use change; (c) Hydrologic and Biogeochemical Legacy Depletion: Data points correspond to mean annual nitrate concentrations measured at the outlet of subwatershed 5 as a function of time since land-use change. The grey shaded area in the figure corresponds to a range of values for the denitrification rate constant (k = 0.24 ± 0.08 y^-1^).

A watershed habitat restoration and agricultural management project was implemented by the United States Fish and Wildlife Service (USFWS) at the Neal Smith National Wildlife Refuge (NSNWR) within the Walnut Creek Watershed (WCW) (52 km^2^) of Jasper County, Iowa (Fig 3a). The project involved conversion of a large portion of the WCW from row-crop agriculture to native prairie and savanna [49,50]. The NSNWR represents one of the first attempts at agricultural land-use conversion towards ecosystem restoration at the watershed scale, and is one of the few sites where water quality has been monitored both in the groundwater directly below the reconstruction and in surface water at multiple scales within the watershed. The site is thus an ideal choice for testing the applicability of the modeling framework introduced in this paper.

The WCW is located in the Southern Iowa Drift Plain landscape region of Iowa, which is characterized by steeply rolling hills and well-developed drainage [51]. The climate of the area is humid and continental. Temperatures in the region vary widely, ranging from average maximum values over 20°C between June and September to less than 0°C in December and January. Annual precipitation averages around 850 mm, with maximum rainfall typically occurring in the months of May and June.

In 1990, the land cover in the watershed was predominantly agricultural, with 70% of the area being covered by row crops. From 1990 to 2005, row-crop cover throughout the watershed was decreased from 70% to 55% as a part of prairie conversion efforts. In subwatershed WNT5 (7.9 km^2^), which is our focus herein, the row crop cover was decreased from 77% to 46%, and surface water quality was monitored subsequently over a period of 13 years [49]. Trajectories for groundwater nitrate concentrations throughout the conversion area were established based on water sampling from 19 monitoring wells across a chronosequence of sites, as indicated by the data points in Fig 3b [52]. For the chronosequence work, sites were selected to represent a conversion time series, with three of the sites still under row crop and the rest having been converted 2–13 years prior to the sampling date. Nitrate concentrations were measured at the outlet of subwatershed 5 and were used to calculate mean annual concentrations (Fig 3c). This site thus provided us with an opportunity to test the ability of the model to capture the dynamics in biogeochemical legacy depletion based on groundwater data, and combined hydrologic and biogeochemical legacy depletion based on surface water data.

#### 3.1B Model Parameters

As described in Section 2.1, our model assumes changes in source zone concentrations over time after conversion from row crop to grassland as a function of both the depletion of legacy soil organic nitrogen (SON) and annual recharge rates to groundwater. To model the biogeochemical legacy dynamics within the source zone, legacy SON is considered to exist within the soil profile to a depth of 1 m at a quantity of 100 kg/ha over baseline (pre-agricultural intensification) levels. This value is a conservative estimate based on N accumulation rates of 6 kg/ha-y for a soil depth of 0–100 cm observed across Iowa under intensive agricultural practices over a period of 70 years (1940–2010) [53], with an assumption that approximately 75% of this accumulation would remain protected via both physical and chemical stabilization mechanisms [54] and that the remaining 25% would be in a readily mineralizable form. Initial NO_3_-N concentrations in the source zone are assumed to be 15 mg/L based on a reported range of 10–20 mg NO_3_-N/L in tile drainage and groundwater under corn-soybean rotations [55,56].

The groundwater travel time distribution for the WCW was determined using a MODFLOW model that was calibrated against measured groundwater elevations at 84 monitoring wells within the site [57–59]. A particle-tracking simulation revealed an exponential travel time distributions for the row-cropped area of the WCW (~ 70% of the watershed is row-cropped) [58]. Reported data on prairie plantings [49] and spatial maps of watershed travel times created using the MODFLOW model [59] demonstrated that the pattern of land-use conversion for WNT5 was predominantly random, which is consistent with our general understanding of land-use shifts for restoration being driven more strongly by the availability of land parcels than design of an optimal land-use change scheme for maximization of water quality benefits. We use a denitrification rate constant (k) varying over a range of 0.24 ± 0.08 y^-1^, which corresponds to a reported range of denitrification rate constants for shallow aquifers with upland surficial geology characterized by glacial outwash and till [60], as is found at the Walnut Creek site. Other parameters used in the model are included in Table 1.

**Table 1:** Model Parameters for the Walnut Creek Watershed.

Model parameters	Walnut Creek Values
Initial Source Zone Nitrate Concentration	15 mg NO_3_-N/L
Initial Mass of Legacy SON	100 kg/m^2^
Legacy N depletion rate constant (λ)	0.16 y^-1^
Denitrification Rate (k)	0.24 ± 0.08 y^-1^
Mean Travel Time (μ)	21.6 y
Mean Annual Recharge (Q)	129.5 mm/y
Soil Saturation (s)	0.5
Soil Porosity (n)	0.3
Fractional Land Area Converted	0.41

### 3.2 Metrics for evaluating concentration reduction benefits

To quantify the concentration reduction benefits achievable at a specified time interval (t) based on a given percent land-use change, we have developed the *CR*_*t*_ metric, defined as:

$$CR_{t}=1-\frac{c_{ac}}{c_{bc}}$$ (13)

For the special case of concentration reductions at very long times *t*→∞, thus representing the maximum benefit that can be achieved by land-use conversion, the *CR*_*inf*_ metric is used, defined as:

$$CR_{\inf}=1-\lim_{t\to \infty }\frac{c_{ac}}{c_{bc}}$$ (14)

## Results and Discussion

The objective of the present study was to develop a framework to quantify catchment-scale time lags based on both biogeochemical and hydrologic nutrient legacies in intensively managed catchments. Our first intent was to develop a set of analytical equations to quantify water-quality benefits, taking into account both soil legacy accumulation and denitrification dynamics along the groundwater pathway. Our results, based on idealized scenarios of land-use conversion, are compared with results related to actual patterns of land-use conversion in the Walnut Creek watershed. Additionally, our intent was to utilize the analytical equations to explore concentration-reduction benefits associated with different spatial patterns of land-use conversion, and thus to further our understanding of both natural and anthropogenic controls on such benefits and any associated time lags. Benefits are gauged in terms of (1) the relative magnitude of the watershed chemical response to the cropland conversion and (2) the arrival time of the response at the outlet. These results are used to establish an optimization framework that clarifies tradeoffs between the land area taken out of row-crop production and the time required to achieve desired concentration-reduction benefits.

### 4.1 Model Validation: The Walnut Creek Case Study

We first applied our model, which takes into account both biogeochemical legacy and the groundwater denitrification dynamics, to the Walnut Creek watershed. The temporal trajectory of source-zone nitrate concentrations *C*_*s*_(*t*) in land parcels that had undergone conversion from row-crop to grassland was modeled using Eqs 4 and 5. A legacy depletion rate constant, λ, of 0.16 per year was able to capture the observed trends in the groundwater chronosequence data described in Section 3.1A and as shown in Fig 3b. The time required to achieve a 50% concentration reduction in the source zone was found to be approximately 5 years, while a 95% concentration reduction in the source zone corresponded to a lag of approximately 19 years.

The concentration trajectory at the catchment outlet was then derived as a function of *C*_*s*_(*t*), the groundwater travel time distribution, the denitrification rate constant, and the pattern of land-use change, following Eqs 10, 11 and 12. The exponential travel time distribution derived from the MODFLOW model (mean travel time μ = 21.6 years) was used long with an assumption of a random pattern of land-use conversion (see Methods 3.2), to model three different scenarios for trajectories of water-quality change after land conversion for the WNT5 subwatershed (Fig 3c).

The first scenario (S1) parallels the approach used by Schilling et al. [49], assuming the presence of hydrologic legacy but no biogeochemical legacy, with no denitrification occurring along the groundwater pathway. Accordingly, there is nitrate dissolved in groundwater that continues to arrive at the outlet over a defined time period (as a function of the groundwater travel time distribution) after land-use conversion, thus shaping the outlet concentration trajectory. The second scenario (S2) maintains the same assumptions as those utilized in S1, but adds denitrification in the saturated groundwater. Under this scenario, nitrate concentrations decrease as they travel from the source zone to the outlet, as nitrate is reduced to N_2_ or N_2_O and leaves the system in a gaseous form. Both S1 and S2 assume that groundwater concentrations in the source zone, beneath the land parcels for which the land-use shift has occurred, drop immediately from C_s_ to 0, and that the observed concentration trajectory at the outlet is only a function of dynamics along the groundwater flow pathways.

In contrast, the third scenario (S3) takes into account both denitrification and the presence of biogeochemical legacy in the source zone. The *C*_*s*_(*t*) function in the model, as shown in Fig 3b, is convoluted with the groundwater travel time distribution following Eq 1 to estimate the concentrations at the catchment outlet. As can be seen in Fig 3c, the base case scenario S1, after year 3, generally overestimates the concentration at the outlet. Conversely, the S2 scenario, which takes into account denitrification dynamics, consistently underestimates the achieved concentrations, even when considering the full range of possible values for the denitrification rate constant (k). However, with S3’s combined consideration of both denitrification and biogeochemical legacy, there is a close match between predicted concentration reductions and the observed data for WNT5. In particular, S3 captures the time lag between the initial land conversion and the first observed drop in concentrations at year 4.

The model thus provides a parsimonious way of describing the concentration trajectory, both at the parcel in which land use change has occurred, and at the catchment outlet. Although in the present study we used the more computationally intensive MODFLOW/MODPATH approach to estimate the groundwater travel time distribution, previous work [58] suggests that a simple GIS-based approach, which uses the land surface as a surrogate for the water table, can be used to construct the travel-time distribution. The latter method is based on readily available DEM data and hydraulic conductivity values, which can be obtained at the local scale from soil databases and at larger, regional scales from recently constructed global maps of near-surface permeability [61].

### 4.2 Concentration Reduction as a Function of Spatial Patterns of Land-Use Change

**Fig 4:**
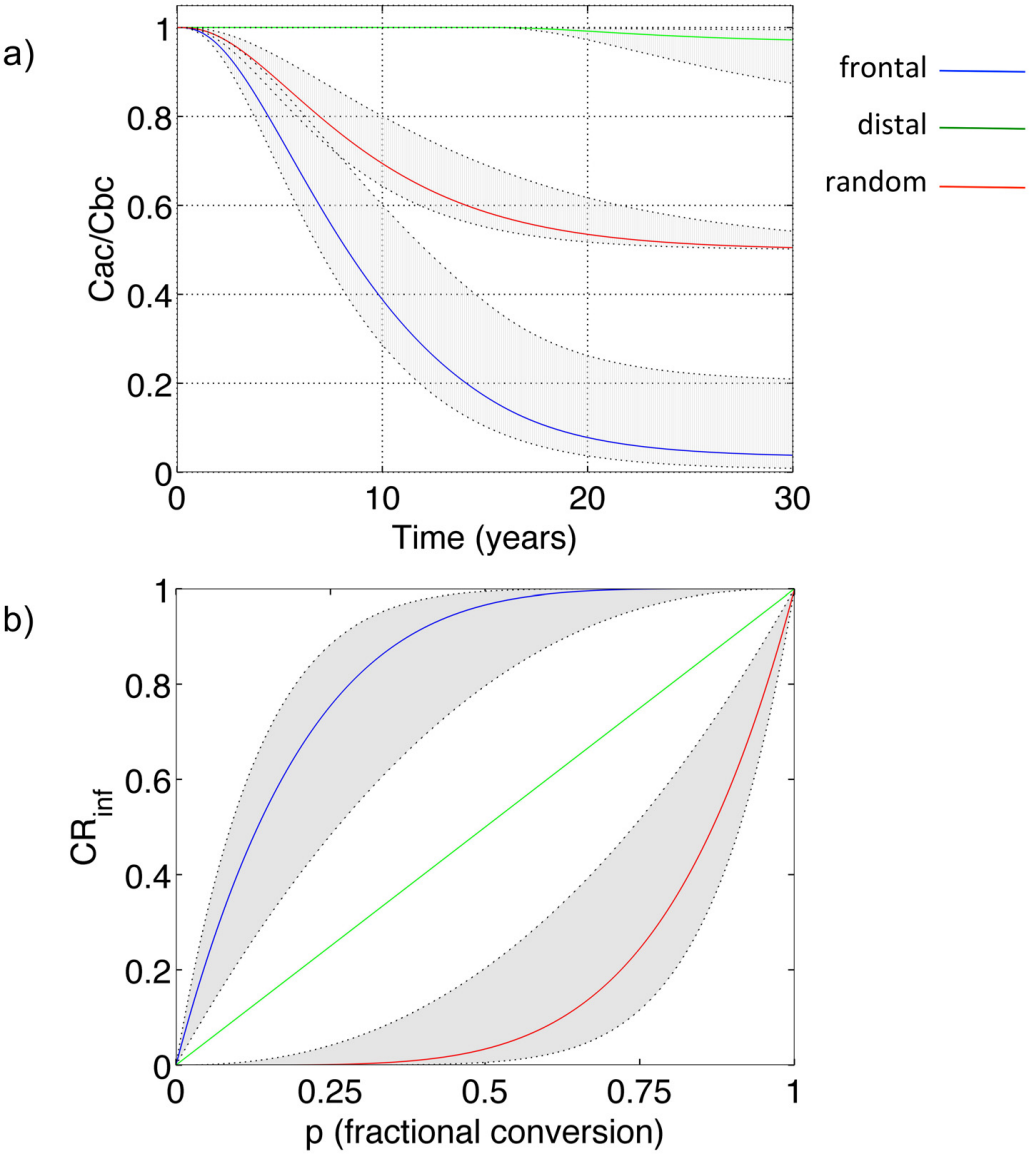
Normalized concentration reduction trajectories under different patterns of land-use change. (a) Normalized concentration trajectories at the catchment outlet plotted as a function of time (years) after land-use change for the frontal, random and distal patterns of conversion; fractional land-use conversion p = 0.5; (b) Concentration reduction fraction at infinite time as a function of land use conversion fraction p. In both figures, k = 0.18 ± 0.12, which corresponds to a range of “moderate” denitrification rates (Tesoriero et al. 2011). Other parameters used are lambda = 0.23 y^-1^ and μ = 21.6 y. A 1:1 relationship between CR_inf_ and p, with no dependence on the k values is apparent for the random truncation.

We next utilized our analytical equations to explore concentration-reduction benefits associated with different spatial patterns of land-use conversion. The temporal trajectories for the outlet concentration after conversion (50% land-use conversion, k = 0.18 ± 0.12 y^-1^) normalized to the mean concentration before conversion are presented in Fig 4a. The frontal conversion leads to the fastest response and the distal the slowest, with the random somewhere in the middle. For both frontal and random truncation of the groundwater travel time distribution, partial benefits are immediately realized at the watershed outlet, but for a distal truncation there is a time lag between the implementation of change and the start of benefit realization. This time lag corresponds to the minimum travel time of the altered land-use parcels, *F* ^*-1*^(1-*p*), and is a function of both the groundwater travel time distribution characteristics and the fractional land-use change. In the modeled scenario, this time lag is approximately 16 years, whereas in the frontal and random scenarios concentration reductions of approximately 85% and 43%, respectively, have already been achieved at 16 years after conversion (Fig 4a).

Importantly, not only the time required to achieve a desired concentration reduction, but also the maximum achievable concentration reduction at infinite time (*CR*_*inf*_), differs according to the spatial pattern of conversion. This spatial dynamic is captured in Fig 4b, in which *CR*_*inf*_ values are plotted as a function of the fractional land-use conversion, p, for frontal, distal, and random conversion scenarios. As can be seen in the figure, the frontal pattern of intervention provides the greatest maximum concentration reduction benefit at all percentages of landscape conversion, with the greatest difference between the frontal and distal scenarios occurring under the 50% conversion scenario. The relatively low *CR*_*inf*_ values for the distal scenarios demonstrate that even at very moderate denitrification rates (k = 0.18 ± 0.12 y^-1^), land parcels with relatively greater travel times make very little contribution to stream nitrate concentrations. Accordingly, conversion of those parcels will have virtually no impact on nitrate concentrations at the watershed outlet.

In contrast, it should be noted that a random pattern of intervention provides a 1:1 concentration reduction benefit. In other words, when interventions are applied randomly throughout a watershed, a 20% conversion of watershed area will result in a 20% reduction in concentration. Mathematically, this 1:1 relationship between land-use conversion and water quality benefits arises due to the property by which a probability distribution created by taking a large enough random sample from any frequency distribution will have the same attributes as the original distribution. It should also be noted that though a range of *CR*_*inf*_ values is obtained for the frontal and distal conversion scenarios, based on the range of denitrification rate constants, the values for the random scenario are not a function of this rate constant and therefore do not deviate from the 1:1 relationship between land-use conversion and concentration reductions.

### 4.3 Concentration reductions as a function of natural and anthropogenic controls

#### 4.3A Concentration Reductions at Infinite Time

**Fig 5:**
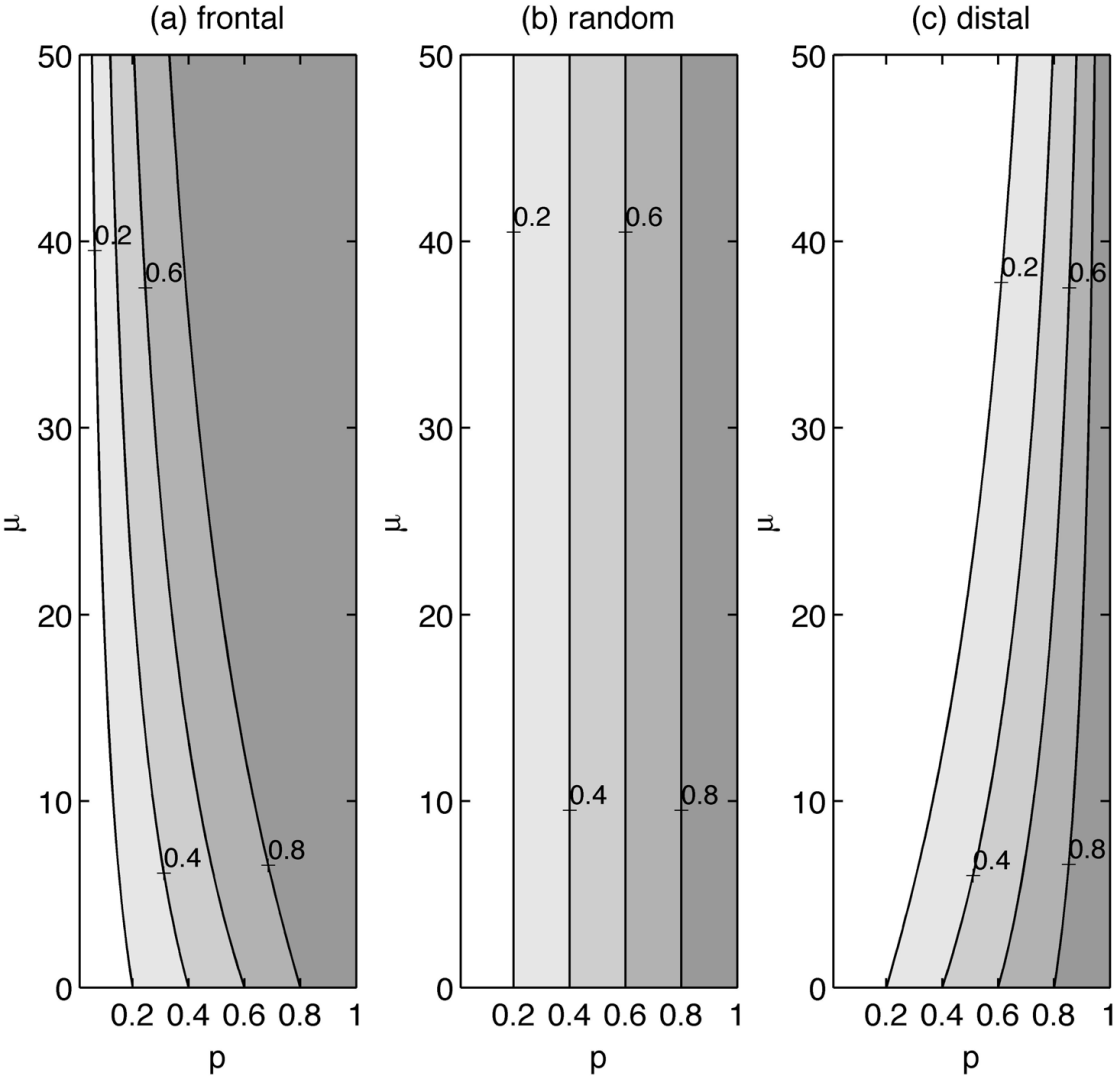
Maximum normalized concentration reduction (CR_inf_) contours plotted as a function of the fractional land-use conversion p and mean watershed travel time *μ*. Contours are plotted for the (a) frontal, (b) random and (c) distal truncation scenarios (k = 0.06 y^-1^, *λ* = 0.16 y^-1^).

In the above sections, we have focused on concentration-reduction benefits corresponding to one, unique travel time distribution (exponential with *μ* = 26 years). In order to understand how such benefits vary as a function of the travel time distribution, we have plotted contours of maximum concentration reductions (*CR*_*inf*_) along a continuum of values for both the mean travel time (*μ*) and the fractional area within a watershed being removed from row-crop production (*p*) (Fig 5). Three different plots are presented (5a, 5b,5c), corresponding to the frontal, random and distal, patterns of intervention, respectively. For a particular watershed (characterized by its *μ* value), the *CR*_*inf*_ benefit achieved for a specified fractional land-use change (*p*) is equal to *p* for the random truncation scenario (Fig 5b), greater than *p* for the frontal scenarios (Fig 5a) and less than *p* for the distal scenarios (Fig 5c). For a particular conversion fraction *p*, the concentration reduction benefits increase with increasing mean travel times for the frontal truncation scenario, while they decrease for the distal truncation and remain invariant with *μ* for the random truncation.

#### 4.3B Concentration Reductions at Specified Times

**Fig 6:**
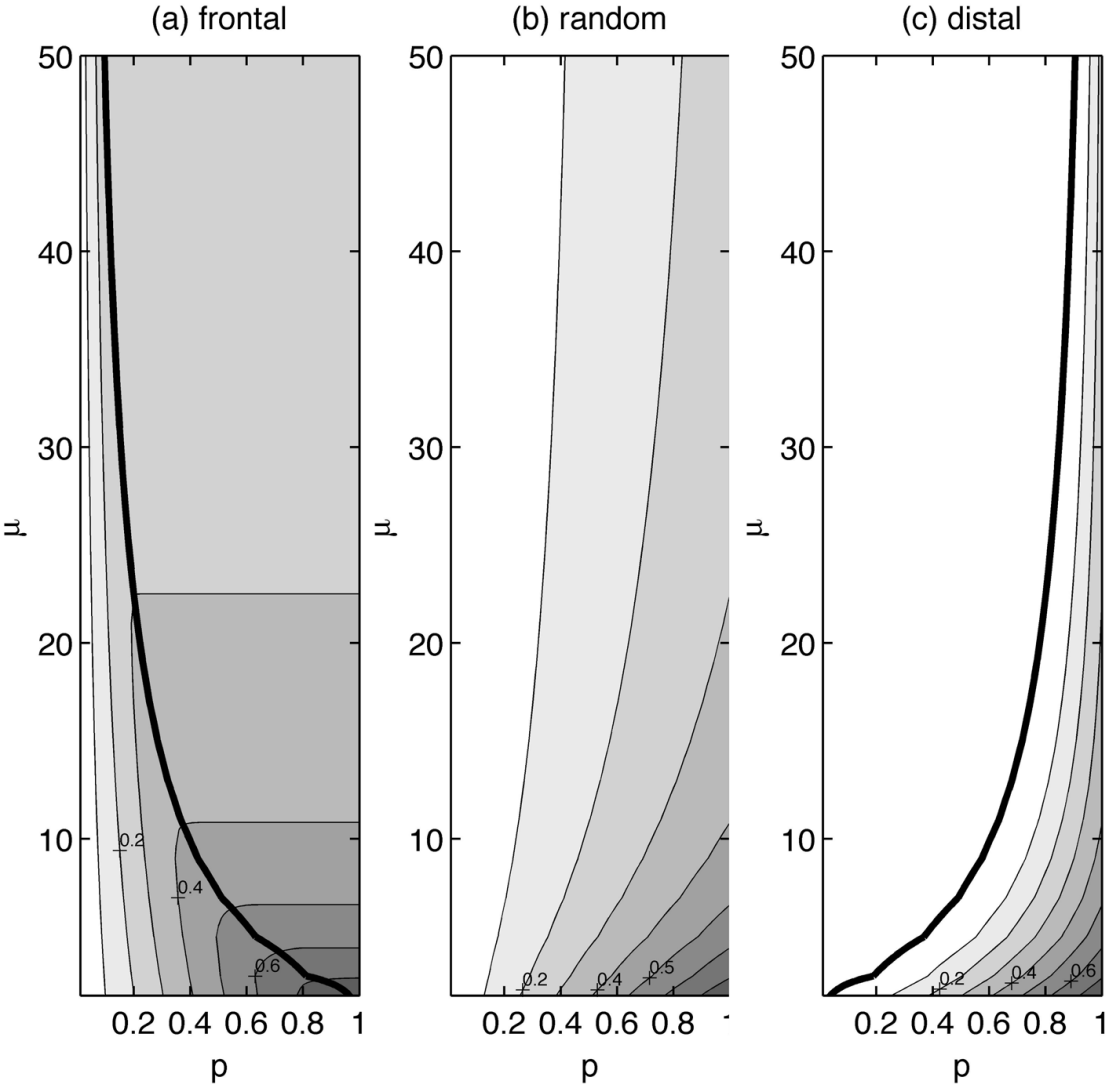
Normalized concentration reduction contours at t = 5 years (CR_5_) plotted as a function of the fractional land-use conversion p and mean watershed travel time *μ*. Contours are plotted for the (a) frontal, (b) random and (c) distal truncation scenarios (k = 0.06 y^-1^, *λ* = 0.16 y^-1^).

The above analysis describes concentration reduction benefits occurring at infinite time. Decisions regarding land management, however, are not made based on hypothetical benefits achieved at infinite times, but on realistic concentration reductions achievable within specific time frames. In Fig 6, we show the concentration reduction profiles achievable 5 years after landscape conversion (*CR*_*5*_ values) as a function of *p* and *μ*.

With a frontal conversion (Fig 6a), it can be seen that 5 years after conversion (*t*_*d*_ = 5), the concentration reductions for a particular watershed (characterized by a *μ* value) increase with increases in *p* up to a point, and then become invariant with *p*. Beyond this point, further land-use conversion in this watershed results in no additional stream water quality benefits within the 5-year period, as land being converted beyond this threshold point has an associated travel time greater than 5 years, and thus has no impact. As *μ* increases, this threshold point shifts to the left, implying that the threshold is crossed at lower and lower *p* values. This trend occurs because for larger watersheds, a 5-year threshold is only a small percentage of its overall area, and thus benefits cease beyond a relatively small value of *p*. The thicker line connecting the threshold points thus divides the plot are into two zones, one where benefits are still being realized and another for which benefits have ceased, with the line being mathematically denoted by F^-1^(p) = t_d_.

It is important to understand the existence of this threshold when designing restoration schemes. For example, land managers working in a watershed with a mean groundwater travel time of 15 years may be under pressure to reduce stream NO_3_^-^ concentrations by 50% over a period of 5 years. Knowing that proportionally greater benefits will be achieved with a frontal approach to restoration, they begin converting land with the smallest travel times. Fig 6a, however, shows that under these conditions, a concentration reduction of approximately 35% is the greatest benefit that can be achieved within the 5-year period, even if 100% of the land is converted from row-crop production to native prairie.

The results are quite different, however, for the distal and random conversion scenarios. With a distal conversion, the threshold line is the zero-benefit contour (the heavy dark line in Fig 6c), such that to the left of this line, no concentration reduction benefit can be achieved. Compared with the frontal scenario, it can be seen that a distal approach provides much poorer outcomes, requiring a much greater conversion area and/or a much longer time periods to see results. Assuming the same scenario as above, with a mean groundwater travel time of 15 years, land managers would be forced to convert approximately 90% of the watershed from row-crop to prairie to achieve the same 30% concentration reduction that could be achieved with an approximately 20% conversion area under the frontal approach. Finally, with a random approach, as shown in Fig 6b, concentration benefits scale continuously with *p* and *μ*. Additionally, as was seen in the *CR*_*inf*_ plots in Fig 5, a random conversion approach provides poorer concentration reduction outcomes than the frontal approach, but better outcomes than the distal approach.

#### 4.3C Time Lags and Tradeoffs

**Fig 7:**
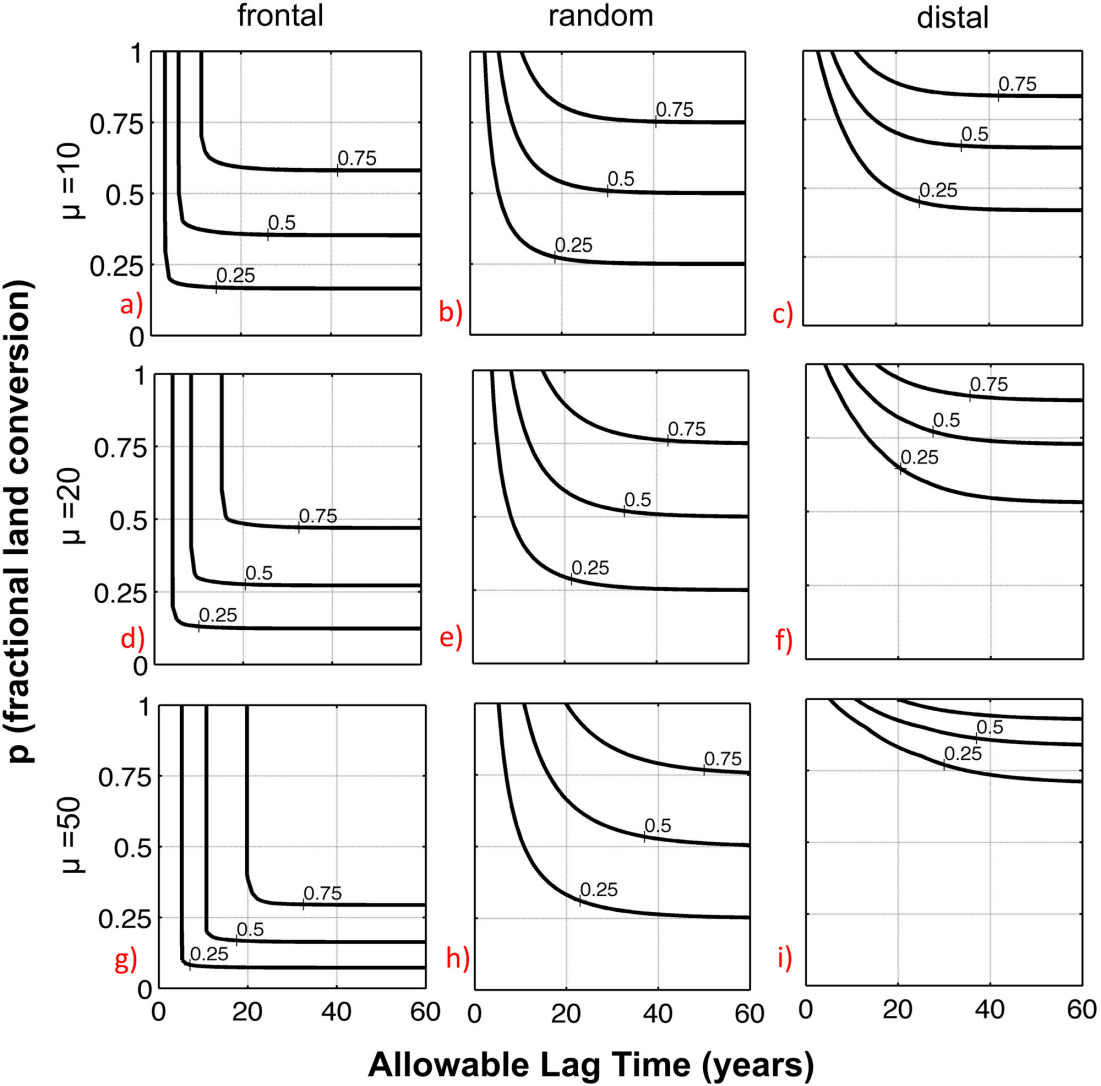
Normalized concentration reduction contours at infinite time as a function of the allowable lag time and the fractional land-use conversion. The three rows represent different watershed mean travel times, while the three columns represent frontal, random and distal patterns of land-use change (k = 0.06 y^-1^, *λ* = 0.16 y^-1^).

With an understanding of catchment-scale time lags, an optimization approach can be developed to clarify the tradeoffs involved with achieving a specified concentration reduction benefit. In our case, conversion of land in row-crop agriculture to native prairie can be understood within the framework of two competing objectives. Objective 1 (O1) is to achieve specified nitrate concentration reduction goals within a desired time frame. Objective 2 (O2) is to minimize both societal and individual farmer costs associated with implementation of environmental interventions while still meeting concentration reduction goals. Fig 7 provides a visualization of Pareto-optimal fronts for these conflicting objectives, with the contour lines representing progressively greater concentration reduction goals, from 25 to 75% reduction. In the figure, the x-axes correspond to the time in years after land conversion from row-crop to native prairie necessary for the concentration reductions to be realized (O1), while the y-axes correspond to the economic costs of land converted (O2), with the fractional land are converted (*p*) serving as a proxy for costs incurred. The three columns correspond to the frontal, random and distal approaches to intervention, with each resulting in its own family of optimized values for land conversion and time required to see the specified concentration reduction benefit. Watersheds with different travel time distributions are also represented here, with rows 1, 2 and 3 corresponding to mean travel times of 10, 20 and 50 years, respectively.

As can be seen in the figure, to achieve progressively greater concentration reduction goals, tradeoffs are necessary between the percent land converted and the time to concentration reduction. For example, if a random approach is taken to carrying out land conversion, as is typical in most watersheds, and a 50% concentration reduction is desired, the time required to achieve the desired concentration benefit ranges from approximately 8 to 30 years (*μ* = 10 years) (Fig 7c). If Objective 1 is prioritized, to give the fastest possible response time, a more than 90% conversion away from row-crop must be carried out. Conversely, if Objective 2 (cost) is prioritized, to maintain the maximum land in production, a 50% conversion is required, with the understanding that there will be a multi-decade time lag between conversion and fully meeting CR goals. An optimal compromise position, within the constraints of the random approach, would likely occur somewhere near the midpoint of the contour line, with approximately 70% of area being converted and an 11-year lag time. To further reduce the necessary percent land conversion, and thus to further minimize the economic impact, a frontal approach could be utilized. Although the fastest that a 50% concentration reduction can be achieved with the frontal approach remains at approximately 8 years (Fig 7a), the percent conversion necessary to achieve this reduction within this time period is reduced from 90% to 40%.

Such tradeoffs are also a function of the mean travel time for the watershed. In watersheds of the same size but with different mean travel times, the greater benefits of the frontal approach correlate positively with the travel time, allowing concentration reduction objectives to be achieved with significantly less commitment of resources. For example, in the *μ* = 10 year watershed, a 50% CR requires, at minimum, a close to 40% conversion of land area out of row crop. In contrast, in the *μ* = 50 year watershed the same reduction can be achieved with a 30% conversion over a similar time frame.

In general, it can be seen that the concentration reduction response scales according to both watershed characteristics (mean travel time) and the employed management approach (spatial patterns of intervention), and with such changes the optimal level of intervention can be either more widely or more narrowly defined. If considering only tradeoffs between fractional land-use change (cost) and the time required to achieve target concentrations, the frontal approach provides a more clearly defined optimal intervention, with conversion of additional land area providing little or no additional time advantage beyond a threshold value. In contrast, with a random or more distal approach, the tradeoffs between time and the fractional converted area scale over a wider range of values, thus leading to more room for debate regarding the best path towards achieving concentration reduction goals.

## Summary and Implications

In recent years, there has been great interest and investment of both private and public funds in the implementation of conservation-oriented management practices and other measures to minimize the negative environmental impacts of modern agricultural practices. Such interventions range from the retirement of agricultural land through programs such as the U.S. Conservation Reserve Program, to reductions in fertilizer application and the creation of riparian buffer zones. Interest is also growing in the potential mitigating impacts of large-scale conversion from grain-based cropping systems to the cultivation of perennial biofuel crops such as switchgrass and miscanthus, which have been found to result in reduced nitrate leaching at the plot scale [62]. Although numerous studies have attempted to demonstrate the potential water-quality benefits garnered by implementing such changes [38,63], there has been little acknowledgment of the often long time periods required to achieve such benefits [8]. In addition, most existing models such as SWAT and AGNPS [64,65], which are commonly utilized for agricultural landscapes, do not have an explicit mechanism to either account for such legacies or to predict time lags [66].

In the present work, we have developed a framework that allows for the parsimonious modeling of concentration-reduction benefits over time as a function of spatial patterns of land-use conversion or implementation of conservation measures across the landscape, and the existence of hydrologic and biogeochemical nutrient legacies. Specifically, we have focused on nitrogen, such that biogeochemical legacy refers to sorbed organic nitrogen within the root zone, while hydrologic legacy refers to nitrate dissolved in groundwater. The model was able to capture the concentration dynamics in both shallow groundwater beneath sites undergoing landscape conversion as well in-stream concentrations at the catchment outlet. Our findings indicate that the existence of biogeochemical legacy can more than double the time needed to see meaningful concentration reductions at the catchment scale. In addition, we show that while a random approach to landscape conversion will lead to a 1:1 relationship between land-use conversion and maximum concentration reduction benefits at infinite time, a preferential conversion of land parcels with shorter travel times will lead to both faster recovery times and greater maximum achievable concentration reductions.

Our modeling framework provides a first attempt at fully describing and quantifying the often-ignored time lag in catchment management questions. In its present form, it allows for the quantification of tradeoffs between costs associated with implementation of conservation measures and the time needed to see the desired concentration reductions, thus making it a potentially powerful tool for land management as agricultural pressures on the environment continue to intensify. The analytical framework also makes the approach conducive towards making uncertainty assessments regarding concentration reduction and lag time metrics. In the future, the approach can be further refined by consideration of spatially varying denitrification rate constants, coupled dynamics of denitrification and dissolved organic carbon availability, and by the introduction of hydrologic variability in relation to both rainfall and evapotranspiration dynamics, as they affect the travel-time distribution for the catchment. Such refinements will lead to even further benefits with regard to decision-making support for implementation of conservation measures in intensively managed watersheds.

## References

[1] Howarth RW, Boyer EW, Pabich WJ, Galloway JN. Nitrogen Use in the United States from 1961–2000 and Potential Future Trends. Ambio. 2002;31: 88–96. 12078014 10.1579/0044-7447-31.2.88

[2] Tilman D, Cassman KG, Matson PA, Naylor R, Polasky S. Agricultural sustainability and intensive production practices. Nature. 2002;418: 671–677. 12167873 10.1038/nature01014

[3] Kling CL, Panagopoulos Y, Rabotyagov SS, Valcu AM, Gassman PW, Campbell T, et al LUMINATE: linking agricultural land use, local water quality and Gulf of Mexico hypoxia. Eur Rev Agric Econ. 2014;41: 431–459. 10.1093/erae/jbu009

[4] Diaz RJ, Rosenberg R. Spreading dead zones and consequences for marine ecosystems. Science. 2008;321: 926–929. 10.1126/science.1156401 18703733

[5] Kemp WM, Testa JM, Conley DJ, Gilbert D, Hagy JD. Temporal responses of coastal hypoxia to nutrient loading and physical controls. Biogeosciences. 2009;6: 2985–3008.

[6] Rabalais NN, Diaz RJ, Levin LA, Turner RE, Gilbert D, Zhang J. Dynamics and distribution of natural and human-caused hypoxia. Biogeosciences. 2010;7: 585–619.

[7] Osterman LE, Poore RZ, Swarzenski PW, Senn DB, DiMarco SE. The 20th-century development and expansion of Louisiana shelf hypoxia, Gulf of Mexico. Geo-Mar Lett. 2009;29: 405–414.

[8] Meals DW, Dressing SA, Davenport TE. Lag Time in Water Quality Response to Best Management Practices: A Review. J Environ Qual. 2010;39: 85 10.2134/jeq2009.0108 20048296

[9] Hamilton SK. Biogeochemical time lags may delay responses of streams to ecological restoration. Freshw Biol. 2012;57: 43–57. 10.1111/j.1365-2427.2011.02685.x

[10] Jarvie HP, Sharpley AN, Withers PJA, Scott JT, Haggard BE, Neal C. Phosphorus Mitigation to Control River Eutrophication: Murky Waters, Inconvenient Truths, and “Postnormal” Science. J Environ Qual. 2013;42: 295 10.2134/jeq2012.0085 23673821

[11] Zhang Y-K, Schilling KE. Increasing streamflow and baseflow in Mississippi River since the 1940s: Effect of land use change. J Hydrol. 2006;324: 412–422. 10.1016/j.jhydrol.2005.09.033

[12] Basu NB, Destouni G, Jawitz JW, Thompson SE, Loukinova NV, Darracq A, et al Nutrient loads exported from managed catchments reveal emergent biogeochemical stationarity. Geophys Res Lett. 2010;37 10.1029/2010GL045168

[13] MacDonald GK, Bennett EM. Phosphorus Accumulation in Saint Lawrence River Watershed Soils: A Century-Long Perspective. Ecosystems. 2009;12: 621–635.

[14] Thompson SE, Basu NB, Lascurain J, Aubeneau A, Rao PSC. Relative dominance of hydrologic versus biogeochemical factors on solute export across impact gradients. Water Resour Res. 2011;47 10.1029/2010WR009605 24511165

[15] McMahon PB, Dennehy KF, Bruce BW, Böhlke JK, Michel RL, Gurdak JJ, et al Storage and transit time of chemicals in thick unsaturated zones under rangeland and irrigated cropland, High Plains, United States. Water Resour Res. 2006;42: n/a–n/a. 10.1029/2005WR004417

[16] Baker LA, Hope D, Xu Y, Edmonds J, Lauver L. Nitrogen Balance for the Central Arizona-Phoenix (CAP) Ecosystem. Ecosystems. 2001;4: 582–602. 10.1007/s10021-001-0031-2

[17] Hong B, Swaney DP, Howarth RW. Estimating Net Anthropogenic Nitrogen Inputs to U.S. Watersheds: Comparison of Methodologies. Environ Sci Technol. 2013;47: 5199–5207. 10.1021/es303437c 23631661

[18] David MB, Drinkwater LE, McIsaac GF. Sources of Nitrate Yields in the Mississippi River Basin. J Environ Qual. 2010;39: 1657 10.2134/jeq2010.0115 21043271

[19] Boyer EW, Goodale C, Jaworski N, Howarth RW. Anthropogenic nitrogen sources and relationships to riverine nitrogen export in the northeastern U.S.A. Biogeochemistry. 2002;57: 137–169.

[20] Billen G, Thieu V, Garnier J, Silvestre M. Modelling the N cascade in regional watersheds: The case study of the Seine, Somme and Scheldt rivers. Agric Ecosyst Environ. 2009;133: 234–246. 10.1016/j.agee.2009.04.018

[21] Kroeze C, Aerts R, van Breemen N, van Dam D, Hofschreuder P, Hoosbeek M, et al Uncertainties in the fate of nitrogen I: An overview of sources of uncertainty illustrated with a Dutch case study. Nutr Cycl Agroecosystems. 2003;66: 43–69.

[22] Chen N, Hong H, Zhang L, Cao W. Nitrogen sources and exports in an agricultural watershed in Southeast China. Biogeochemistry. 2008;87: 169–179.

[23] Liu C, Watanabe M, Wang Q. Changes in nitrogen budgets and nitrogen use efficiency in the agroecosystems of the Changjiang River basin between 1980 and 2000. Nutr Cycl Agroecosystems. 2008;80: 19–37.

[24] Janzen HH, Beauchemin KA, Bruinsma Y, Campbell CA, Desjardins RL, Ellert BH, et al The fate of nitrogen in agroecosystems- An illustration using Canadian estimates.pdf. Nutr Cycl Agroecosystems. 2003;67: 85–102.

[25] Schlesinger WH. On the fate of anthropogenic nitrogen. PNAS. 2008;106: 203–208. 10.1073/pnas.0810193105 19118195 PMC2613040

[26] Fowler D, Pyle JA, Raven JA, Sutton MA. The global nitrogen cycle in the twenty-first century: introduction. Philos Trans R Soc B Biol Sci. 2013;368: 20130165–20130165. 10.1098/rstb.2013.0165 23713127 PMC3682749

[27] Zaehle S. Terrestrial nitrogen-carbon cycle interactions at the global scale. Philos Trans R Soc B Biol Sci. 2013;368: 20130125–20130125. 10.1098/rstb.2013.0125 23713123 PMC3682745

[28] Sebilo M, Mayer B, Nicolardot B, Pinay G, Mariotti A. Long-term fate of nitrate fertilizer in agricultural soils. Proc Natl Acad Sci. 2013;110: 18185–18189. 10.1073/pnas.1305372110 24145428 PMC3831475

[29] Haag D, Kaupenjohann M. Biogeochemical Models in the Environmental Sciences. HYLE—International J Philos Chem. 2000;6: 117–142.

[30] Van Meter KJ, Basu NB, Veenstra JJ, Burras CL. The Nitrogen Legacy: Evidence of Soil Nitrogen Accumulations in Anthropogenic Landscapes. Rev.

[31] Sanford WE, Pope JP. Quantifying groundwater’s role in delaying improvements to Chesapeake Bay water quality. Environ Sci Technol. 2013;47: 13330–13338. 10.1021/es401334k 24152097

[32] Bouraoui F, Grizzetti B. Modelling mitigation options to reduce diffuse nitrogen water pollution from agriculture. Sci Total Environ. 2014;468–469: 1267–1277. 10.1016/j.scitotenv.2013.07.066 23998504

[33] Howarth RW, Swaney DP, Boyer EW, Marino R, Jaworski N, Goodale C. The influence of climate on average nitrogen export from large watersheds in the Northeastern United States. Biogeochemistry. 2006;79: 163–186. 10.1007/s10533-006-9010-1

[34] Alam MJ, Goodall JL. Toward disentangling the effect of hydrologic and nitrogen source changes from 1992 to 2001 on incremental nitrogen yield in the contiguous United States. Water Resour Res. 2012;48 24976651

[35] Swaney DP, Hong B, Ti C, Howarth RW, Humborg C. Net anthropogenic nitrogen inputs to watersheds and riverine N export to coastal waters: a brief overview. Curr Opin Environ Sustain. 2012;4: 203–211. 10.1016/j.cosust.2012.03.004

[36] Wellen C, Arhonditsis GB, Labencki T, Boyd D. A Bayesian methodolotical framework for accommodating interannual variability of nutrient loading with the SPARROW model. Water Resour Res. 2012;48 24976651

[37] Chen D, Hu M, Dahlgren RA. A dynamic watershed model for determining the effects of transient storage on nitrogen export to rivers. Water Resour Res. 2014;50: 7714–7730. 10.1002/2014WR015852

[38] Jha MK, Gassman PW, Arnold JG. Water quality modeling for the Raccoon River watershed using SWAT. Trans ASAE. 2007;50: 479–493.

[39] Rabotyagov S, Campbell T, Jha M, Gassman PW, Arnold J, Kurkalova L, et al Least-cost control of agricultural nutrient contributions to the Gulf of Mexico hypoxic zone. Ecol Appl. 2010;20: 1542–1555. 20945758 10.1890/08-0680.1

[40] Lewis DB, Kaye JP, Gries C, Kinzig AP, Redman CL. Agrarian legacy in soil nutrient pools of urbanizing arid lands. Glob Change Biol. 2006;12: 703–709. 10.1111/j.1365-2486.2006.01126.x

[41] Jury WA, Russo D, Streile G, El Abd H. Solute transport through layered soil profiles: Zero and perfect travel time correlation models. Water Resour Res. 1990;26: 13–20.

[42] Maloszewski P, Zuber A. Determining the turnover time of groundwater systems with the aid of environmental tracers: 1. Models and their applicability. J Hydrol. 1982;57: 207–231.

[43] Haitjema HM. Analytic element modeling of groundwater flow [Internet]. Academic Press; 1995. Available:

[44] McGuire KJ, McDonnell JJ. A review and evaluation of catchment transit time modeling. J Hydrol. 2006;330: 543–563. 10.1016/j.jhydrol.2006.04.020

[45] Porporato A, D’odorico P, Laio F, Rodriguez-Iturbe I. Hydrologic controls on soil carbon and nitrogen cycles. I. Modeling scheme. Adv Water Resour. 2003;26: 45–58.

[46] Manzoni S, Porporato A. Soil carbon and nitrogen mineralization: Theory and models across scales. Soil Biol Biochem. 2009;41: 1355–1379. 10.1016/j.soilbio.2009.02.031

[47] Pijanowski B, Ray DK, Kendall AD, Duckles JM, Hyndman DW. Using backcast land-use change and groundwater travel-time models to generate land-use legacy maps for watershed management. Ecol Soc. 2007;12: 25.

[48] Jawitz JW, Fure AD, Demmy GG, Berglund S, Rao PSC. Groundwater contaminant flux reduction resulting from nonaqueous phase liquid mass reduction. Water Resour Res. 2005;41 16173154

[49] Schilling KE, Wolter CF. A GIS-based groundwater travel time model to evaluate stream nitrate concentration reductions from land use change. Environ Geol. 2007;53: 433–443. 10.1007/s00254-007-0659-0

[50] Drobney PM. Iowa prairie rebirth rediscovering natural heritage at walnut creek national wildlife refuge. Ecol Restor. 1994;12: 16–22.

[51] Prior J. Landforms of Iowa [Internet]. Iowa City, IA: University of Iowa Press; 1991 Available: http://sustainableag.unl.edu/pdf/landformsofiowacari.pdf

[52] Schilling KE, Jacobson P. Groundwater conditions under a reconstructed prairie chronosequence. Agric Ecosyst Environ. 2010;135: 81–89. 10.1016/j.agee.2009.08.013

[53] Veenstra JJ. Fifty years of agricultural soil change in Iowa [Internet]. Ames, IA: Iowa State University; 2010. Available:

[54] Six J, Conant RT, Paul EA, Paustian K. Stabilization mechanisms of soil organic matter: Implications for C-saturation of soil. Plant Soil. 2002;241: 155–176.

[55] Li C, Farahbakhshazad N, Jaynes DB, Dinnes DL, Salas W, McLaughlin D. Modeling nitrate leaching with a biogeochemical model modified based on observations in a row-crop field in Iowa. Ecol Model. 2006;196: 116–130. 10.1016/j.ecolmodel.2006.02.007

[56] Strock JS, Porter PM, Russelle MP. Cover cropping to reduce nitrate loss through subsurface drainage in the northern US Corn Belt. J Environ Qual. 2004;33: 1010–1016. 15224938 10.2134/jeq2004.1010

[57] Schilling KE, Jindal P, Basu NB, Helmers MJ. Impact of artificial subsurface drainage on groundwater travel times and baseflow discharge in an agricultural watershed, Iowa (USA). Hydrol Process. 2012;26: 3092–3100. 10.1002/hyp.8337

[58] Basu NB, Jindal P, Schilling KE, Wolter CF, Takle ES. Evaluation of analytical and numerical approaches for the estimation of groundwater travel time distribution. J Hydrol. 2012;475: 65–73. 10.1016/j.jhydrol.2012.08.052

[59] Jindal P. A study of the groundwater travel time distribution at a rural watershed in Iowa: A systems theory approach to groundwater flow analysis. 2010; Available: http://lib.dr.iastate.edu/etd/11454/

[60] Tesoriero AJ, Puckett LJ. O _2_ reduction and denitrification rates in shallow aquifers. Water Resour Res. 2011;47: n/a–n/a. 10.1029/2011WR010471

[61] Gleeson T, Smith L, Moosdorf N, Hartmann J, Dürr HH, Manning AH, et al Mapping permeability over the surface of the Earth. Geophys Res Lett. 2011;38 10.1029/2010GL045565

[62] Smith CM, David MB, Mitchell CA, Masters MD, Anderson-Teixeira KJ, Bernacchi CJ, et al Reduced Nitrogen Losses after Conversion of Row Crop Agriculture to Perennial Biofuel Crops. J Environ Qual. 2012;42: 219–228. 10.2134/jeq2012.0210 23673757

[63] Ng TL, Eheart JW, Cai X, Miguez F. Modeling Miscanthus in the Soil and Water Assessment Tool (SWAT) to Simulate Its Water Quality Effects As a Bioenergy Crop. Environ Sci Technol. 2010;44: 7138–7144. 10.1021/es9039677 20681575

[64] Grizzetti B, Bouraoui F, Granlund K, Rekolainen S, Bidoglio G. Modelling diffuse emission and retention of nutrients in the Vantaanjoki watershed (Finland) using the SWAT model. Ecol Model. 2003;169: 25–38. 10.1016/S0304-3800(03)00198-4

[65] Young RA, Onstad CA, Bosch DD, Anderson WP. AGNPS: A nonpoint-source pollution model for evaluating agricultural watersheds. J Soil Water Conserv. 1989;44: 168–173.

[66] Chen D, Huang H, Hu M, Dahlgren RA. Influence of Lag Effect, Soil Release, And Climate Change on Watershed Anthropogenic Nitrogen Inputs and Riverine Export Dynamics. Environ Sci Technol. 2014;48: 5683–5690. 10.1021/es500127t 24742334

